# 2039: Modifiable risk factors Versus age on developing high predicted cardiovascular disease risk in African Americans

**DOI:** 10.1017/cts.2017.90

**Published:** 2018-05-10

**Authors:** Adam Bress, Lisandro D. Colantonio, John N. Booth, Tanya M. Spruill, Joseph Ravenell, Mark Butler, Amanda J. Shallcross, Samantha R. Seals, Kristi Reynolds, Gbenga Ogedegbe, Daichi Shimbo, Paul Muntner

**Affiliations:** Department of Population Health Sciences, University of Utah, Salt Lake City, UT, USA

## Abstract

OBJECTIVES/SPECIFIC AIMS: Clinical guidelines recommend using predicted atherosclerotic cardiovascular disease (ASCVD) risk to inform treatment decisions. The objective was to compare the contribution of changes in modifiable risk factors Versus aging to the development of high 10-year predicted ASCVD risk. METHODS/STUDY POPULATION: Prospective follow-up of the Jackson Heart Study, an exclusively African-American cohort, at visit 1 (2000–2004) and visit 3 (2009–2012). Analyses included 1115 African-American participants without a high 10-year predicted ASCVD risk (<7.5%), hypertension, diabetes, or ASCVD at visit 1. We used the Pooled Cohort equations to calculate the incidence of high (≥7.5%) 10-year predicted ASCVD risk at visit 3. We recalculated the percentage with a high 10-year predicted ASCVD risk at visit 3 assuming each risk factor [age, systolic blood pressure (SBP), antihypertensive medication use, diabetes, smoking, total and high-density lipoprotein cholesterol], one at a time, did not change from visit 1. RESULTS/ANTICIPATED RESULTS: The mean age at visit 1 was 45.2±9.5 years. Overall, 30.9% (95% CI 28.3%–33.4%) of participants developed high 10-year predicted ASCVD risk. Aging accounted for 59.7% (95% CI 54.2%–65.1%) of the development of high 10-year predicted ASCVD risk compared with 32.8% (95% CI 27.0%–38.2%) for increases in SBP or antihypertensive medication initiation and 12.8% (95% CI 9.6%–16.5%) for incident diabetes. Among participants <50 years, the contribution of increases in SBP or antihypertensive medication initiation was similar to aging. DISCUSSION/SIGNIFICANCE OF IMPACT: Increases in SBP and antihypertensive medication initiation are major contributors to the development of high 10-year predicted ASCVD risk in African Americans, particularly among younger adults.

